# Variability of bothersome menopausal symptoms over time – a longitudinal analysis using the Estonian postmenopausal hormone therapy trial (EPHT)

**DOI:** 10.1186/1472-6874-12-44

**Published:** 2012-12-21

**Authors:** Elina Hemminki, Elena Regushevskaya, Riitta Luoto, Piret Veerus

**Affiliations:** 1 National Institute for Health and Welfare (THL), P.O. Box 30, 00271, Helsinki, Finland; 2 UKK Institute for Health Promotion Research and National Institute for Health and Welfare (THL), P.O. Box 30, 33501, Tampere, Finland; 3 Department of Epidemiology and Biostatistics, National Institute for Health Development, Hiiu 42, 11619, Tallinn, Estonia

**Keywords:** Menopausal symptoms, Duration, Follow-up, Estonia

## Abstract

**Background:**

Very little data are available on the natural course or level of disturbance of vasomotor symptoms among middle-aged women. Using readily collected trial data we studied the persistence of vasomotor symptoms among untreated women.

**Methods:**

In a trial comparing combined hormone therapy to placebo or no treatment (control groups), a cohort of women aged 50–59 at recruitment were followed annually by questionnaires. Women in the control groups (n = 486) were grouped by the number of years followed, with the prevalence and severity of symptoms calculated both cross-sectionally and longitudinally.

**Results:**

About two thirds of the women (67%) reported vasomotor symptoms and half (46%) bothersome symptoms at recruitment. In the cross-sectional analysis, their prevalence declined between recruitment and 1-year follow-up (32% bothersome symptoms) and 2-year follow-up (27%). Thereafter it remained about the same level. In the longitudinal analysis, there was a notable variation in the prevalence of disturbing vasomotor symptoms over time, time entering the study and the compliance to the surveys. In the two groups having most follow-up times, the proportion of women with bothersome symptoms first increased and then decreased.

**Conclusions:**

There was a notable variability in the development of disturbing vasomotor symptoms over time in a selected group of women aged 50–59. Population-based follow-up studies of untreated women would be useful to estimate the symptom burden.

## Background

A number of studies have reported a high prevalence of vasomotor symptoms among middle-aged women [1-5]. In a large review by the National Institutes of Health [6], 14–51% of premenopausal and 30–80% of peri- and postmenopausal women reported hot flashes and night sweats. However, not all women consider hot flushes bothersome [5,7-10]. Sleep disturbances are common among all menopausal women (occurring in 16–42% of premenopausal, 39–47% of perimenopausal and 35–60% of postmenopausal women). In a Finnish population-based study by Hemminki et al. (1995), 28% of women aged 45–64 years reported hot flushes, 38% reported tiredness and 27% different kind of pains (head-, back-, and joint ache). The probability of other symptoms is higher if the woman has vasomotor symptoms [7,11].

Cross-sectional surveys among women of different ages show that hot flushes are most common soon after menopause, but they occur even after 10 years [7,8,12,13]. Some women have not flashes even before menopause [13-15], and among some women they become bothersome only some years after menopause [13].

The consensus around vasomotor symptoms (hot flushes and sweating) and vaginal dryness—though not for other symptoms—is that they are more common during menopause than before or after it [2,6,9,16-18]. Results conflict as to whether or not other complaints are more common around menopause [16] or whether the observed increase is related to age rather than to menopause [2,19].

Very few studies grade the symptoms according to women’s experience of disturbance or what is their patterning [20]. The purpose of this study was to use readily collected data on control women in a hormone therapy trial to study how bothersome vasomotor symptoms are for women and what is their patterning. Originally we had also planned to study their duration, but the available data did not allow that.

## Methods

This is a cohort study based on yearly follow-up questionnaires sent to control women of a trial comparing combined hormone therapy to either placebo or nothing (the control groups) [21]. Women were recruited in Estonia from 1999–2001, and followed by annually mailed questionnaires in 2000–2004. Detailed descriptions of the recruitment, inclusion and exclusion criteria, trial treatment, adherence, follow-up and trial outcomes as well as the content of information leaflets and trial questionnaires have been published elsewhere [21-24]. In the following the main features relevant to this study are described.

A recruitment questionnaire was mailed in 1998–1999 to all women aged 45–64 in two Estonian counties (n = 39 713); the identification was through the Estonian Population Registry. Of the 14 743 women who returned the questionnaire, 6606 respondents were interested in participating in a randomised trial, of which 4295 women were found to be eligible according to the preliminary assessment. The eligibility criteria included age of 50 to 64 and an elapsed time of 12 months or more since the last period at the randomisation stage [21].

A personal invitation was mailed to eligible women in 1999–2001. A total of 2323 women responded to the invitation to visit the trial doctor. After a secondary assessment of eligibility, 1778 women proved to be eligible and were willing to join the trial; 404 women were randomized into the blind hormone therapy (HT) arm, 373 into the placebo arm, 494 into the non-blind HT arm and 507 into the non-treatment arm [21,22]. The 46 women who participated in the pilot study are not included in the present analysis. The women in the blind arms received their drug bottles with coded labels (otherwise identical bottles). Women in the non-blind HT arm had their drug bottles marked with HT.

Only data on women in blind placebo group and non-treatment group aged 50–59 at recruitment (n = 486) are used in this study. Throughout the trial, about 90% of women in the non-treatment group and 95% in the placebo group did not start hormone therapy [24]. The power calculations were made for the main study [21-23], but not for this cohort study based on the trial data.

All participants were mailed annual questionnaires that included questions about their current life situation, health, and use of health services. The response rate was 75% for the first annual survey, 69% for the second annual survey, and 81% of the participants for the final survey mailed at the end of the trial and followed by one reminder. On average, the final survey was completed 3.6 years after the recruitment. Each of the survey questionnaires had a list of 18 symptoms experienced in the previous two weeks and an additional question asking women to specify which they had found bothersome. The questions concerning symptoms that were related to menopause (translated into English) are given in Additional file 1: Appendix 1.

All participants gave written informed consent. The study protocol was approved by the Committee of Medical Ethics in Tallinn, Estonia and by the Ethics Committee of the University Clinic of Tampere, Finland.

### Analysis

The non-compliant women (about 9% of women used hormone therapy for some time during the trial in the control group) were included in this analysis. Results were analyzed separately for those aged <55 years and 56–59 years old at recruitment, and as a combined age group. For the longitudinal analysis, women were grouped into 6 groups by the timing and order of follow-up surveys they completed. The aim was for surveys to be completed at 12-month intervals. However, postal dispatch was done according to groups and the time could be somewhat more or less than 12 months. Furthermore, some women answered after some delay. Thus, we first calculated for each woman the months between the recruitment and the different surveys using the date of filling in the questionnaire. We then grouped the surveys as follows: 6–18 months = 1-year follow-up, 19–28 months = 2-year follow-up, 29–42 months = 3-year follow-up, 43–59 months =4-year follow-up. Women who left one or more follow-up surveys unanswered formed a separate group (intermittent surveys).

We classified women into those who had or had not had symptoms in the following symptom groups: 1) vasomotor symptoms (cold sweats, hot flashes), 2) psychological symptoms (feeling blue or depressed, irritability), 3) pains (backache, headache, stomach ache, stiffness or joint pain). If a woman mentioned at least one of the symptoms from a group, the woman was categorized as having the symptoms of that group. If at least one of these symptoms was said to be bothersome, the woman was categorized as having bothersome symptoms of that group.

## Results

Table 1 gives the background characteristics of the women at recruitment. The mean age at recruitment was 55 years (SD 2.5), most were married or cohabiting, were well educated, and were urban dwellers. The two age-groups were very similar by the studied background characteristics.

**Table 1:** Description of the women at recruitment, %

	**<55**	**56–59**	**Total**
(number)	182	304	(486)
Age, years			
50–52	43		16
53–54	57		21
55–57		54	34
58–59		46	29
Marital status			
Married or cohabiting	63	63	63
Divorced or widow	29	32	30
Single or missing	8	6	7
Education			
primary/basic	7	9	8
secondary	35	28	31
vocational	30	27	28
university	28	36	33
Urban	73	74	73
Total	100	100	100

Table 2 gives the prevalence of vasomotor symptoms according to a cross-sectional analysis. At the time of recruitment, about two thirds of the women reported vasomotor symptoms, and about a half regarded them as bothersome. In the survey a year later, less (53%) of women reported vasomotor symptoms and a third found them bothersome. In the surveys 2–4 years later, the proportions reporting symptoms were lower (around 40%), while a fourth of the women reported them being bothersome. The prevalence of women reporting bothersome symptoms declined by 29% from recruitment to the 1-year follow-up and by 41% to the 2-year follow-up. Thereafter the prevalence of symptoms remained at roughly the same level.

**Table 2:** Proportions (%) of women with vasomotor symptoms and bothersome symptoms^1^ in past two weeks, at each survey (cross-sectional analysis)

	**Recruitment**	**1-year**	**2-year**	**3-year**	**4-year**
**Women <55**					
number of women	182	129	124	64	22
(mean age)	(52.7)	(53.8)	(54.9)	(55.7)	(56.4)
any	75.8	54.3	41.9	42.9	31.8
bothersome^2^	50.0	34.1	29.0	34.4	18.2
(of vasomotor^3^)	(66.0)	(62.8)	(69.2)	(80.2)	(57.2)
**All women (50–59)**					
Number of women	486	352	330	204	66
(mean age)	(57.1)	(56.6)	(57.7)	(58.8)	(59.8)
any	67.3	53.1	43.0	42.9	45.5
bothersome^2^	45.7	32.4	27.0	24.0	24.2
(of vasomotor^3^)	(67.9)	(61.0)	(62.7)	(55.9)	(53.2)

^1^ sweating and/or hot flashes.

^2^ at least one of the symptoms was bothersome, % of women.

^3^ women with bothersome vasomotor symptoms of women with any vasomotor symptoms.

Table 3 gives the results of the longitudinal analysis in which the same women were followed from one survey to another. There were some differences by the number of follow-ups (how early or late the woman was recruited) as well as by compliance with the surveys (did she skip some of the surveys, group “Intermittent surveys” in Table 3). In the groups with only three or two measurements or intermittently, the proportion declined over time. In the group having most follow-ups, the proportion of women with bothersome symptoms first increased and then decreased. In the group with three follow-ups, the proportion first declined, and then stayed about the same. Some women had symptoms variably (i.e. having one or more measurements without symptoms and then again reporting symptoms). Younger women did not systematically have more bothersome symptoms than older women.

**Table 3:** Proportions (%) of women with bothersome vasomotor symptoms in past two weeks over time, by the number of follow-ups women had (longitudinal analysis)

	**Recruitment**	**1-year**	**2-year**	**3-year**	**4-year**
**Women <55 (n = 182)**					
4 follow-ups (n = 11)	27.3	54.5	36.4	27.3	9.1
3 follow-ups (n = 44)	52.3	31.8	36.4	38.6	-
2 follow-ups (n = 48)	45.8	33.3	21.0	-	-
1 follow-up (n = 16)	37.5	25.0	-	-	-
(% decline)		(33.3)	(100)	-	-
Intermittent surveys^1^ (n = 31)	48.4	44.4	25.0	9.1	20
Only recruitment (n = 32)	68.8	-	-	-	-
**All women (50–59) (n = 304)**					
4 follow-ups (n = 47)	40.4	51.1	31.9	27.7	23.4
3 follow-ups (n = 121)	39.7	24.0	27.3	22.3	-
2 follow-ups (n = 121)	42.1	34.7	23.1	-	-
1 follow-up (n = 40)	40.0	30.0	-	-	-
(% decline)		(25.0)	-	-	-
Intermittent surveys ^2^ (n = 74)	54.1	36.8	17.6	16.4	6.8
**Total (n = 486)**	45.7	32.4	27.0	24.0	24.2
Only recruitment (n = 83)	57.8	-	-	-	-

^1^ women participating in the specific survey included, n varies between 6–16.

^2^ women participating in the specific survey included, n varies between 19–74.

Comparing the proportions of women with bothersome symptoms at various times to those in the cross-sectional analysis of Table 2 reveals relatively similar percentages.

For comparison, we also studied psychological symptoms (depression and/or irritability) and pains (head, back and/or joint pains; stomach) in the cross-sectional analysis (Data not shown). The prevalence of bothersome psychological symptoms (depression or irritability) declined notably (by about half) from recruitment to the 3-year follow-up, but there was very little change in the prevalence of women reporting bothersome pains.

### Comments

About two thirds of the women in this selected group of women reported vasomotor symptoms and about half reported them being bothersome symptoms. There was a notable variation in the development over time in the prevalence of bothersome symptoms. The development over time was related both to the timing of entering the study, and the compliance with answering the surveys. Our sample size was too small to analyze this in relation to background characteristics to explain the variation.

The results of the longitudinal analysis (comparing the same women over time) and those of the cross-sectional analysis showed relatively similar results. Thus, we can interpret the cross-sectional data as generally summing up the experience of all women throughout the follow-up, even though different women answered in different surveys.

We had originally planned to make a longitudinal analysis using time-to-event calculations (event being the end of symptoms) and estimate the duration of vasomotor symptoms. However, we did not have reliable data of the duration of symptoms before recruitment and complete measurements were not available for all women. Furthermore the numbers of women in early menopause were few.

These complications of data led us to the crude analysis used in the study. However, our analysis was good enough to show the variability between women, and suggest the need for larger follow-up studies to describe the natural course of menopausal symptoms. To estimate the duration of symptoms among unselected women and to estimate the burden of symptoms related to menopause, one would need a follow-up since menopause in a population not using hormone therapy.

Our study women are not a representative sample of women, but women who were motivated to join a preventive drug trial. A comparison of women wanting and not wanting to join the trial showed that the studied menopausal symptoms were four times more common among those who initially expressed an interest than among those who were not interested at the time of asking [25]. Thus, at a population level, the prevalence of symptoms is much lower, but we know nothing of whether the disturbance and variability among those who have symptoms is the same as in our trial.

For comparison, we studied bothersome psychological symptoms and pains in the cross-sectional analysis. The psychological symptoms were less common, but otherwise behaved as vasomotor symptoms; there was very little change in the prevalence of women reporting bothersome pains. If a longitudinal study on menopausal women were to be made, other non-menopausal symptoms should also be studied.

As the overall balance of effects of postmenopausal hormone therapy (HT) on chronic diseases is not positive (at the population level) [26] the use of HT is argued largely on the basis of symptom relief. As the current knowledge of the frequency or level of disturbance of symptoms is patchy [14], population-based follow-ups on the natural course of menopausal symptoms are clearly needed. Even though such averaged information does not accurately predict the fate of individual women, it would be useful for women when they are trying to decide whether or not to use HT. In the past such research was difficult as HT was very commonly used. With the current knowledge on the mixed effect on diseases, it might be feasible to find a representative cohort of new menopausal women without HT for follow-up studies.

## Conclusion

There was a notable variability in the development of disturbing vasomotor symptoms over time in a selected group of women aged 50 -59. Population-based follow-up studies of untreated women would be useful to estimate the symptom burden.

## Competing interests

The authors declare that they have no competing interests.

## Authors’ contributions

EH originated the idea, planned the analysis and prepared the draft manuscript. ER carried out the analysis and commented the manuscript. RL commented the manuscript. PV participated in the designing of the analysis and commented the manuscript. All authors read and approved the final manuscript.

## Pre-publication history

The pre-publication history for this paper can be accessed here:

http://www.biomedcentral.com/1472-6874/12/44/prepub

## Supplementary Material

Additional file 1 **Appendix 1.** Key questions/ questions on symptoms. Click here for file
