# Supplementary material for: Variability of bothersome menopausal symptoms over time – a longitudinal analysis using the Estonian postmenopausal hormone therapy trial (EPHT)
Source: BMC Womens Health. 2012 Dec 21;12:44. doi: 10.1186/1472-6874-12-44 (PMC3542280; doi:10.1186/1472-6874-12-44)
Supplement: Additional file 1 — Appendix 1. Key questions/ questions on symptoms. [file 1472-6874-12-44-S1.doc]

Appendix A. Appendix 1

A.1. Key questions/questions on symptoms

Did you have any of the following symptoms in last two weeks?

|  | **No** | **Yes** | **I do not remember** |
| --- | --- | --- | --- |
| dizziness (1) | 1 | 2 | 3 |
| tiredness (2) | 1 | 2 | 3 |
| diarrhoea or constipation (3) | 1 | 2 | 3 |
| irritability (4) | 1 | 2 | 3 |
| constant cough (5) | 1 | 2 | 3 |
| depression (6) | 1 | 2 | 3 |
| backache (7) | 1 | 2 | 3 |
| stomach pain (8) | 1 | 2 | 3 |
| headache (9) | 1 | 2 | 3 |
| cold sweats (10) | 1 | 2 | 3 |
| joint/muscle ache (11) | 1 | 2 | 3 |
| shortness of breath (12) | 1 | 2 | 3 |
| hot flashes (13) | 1 | 2 | 3 |
| sore throat (14) | 1 | 2 | 3 |
| sleeplessness (15) | 1 | 2 | 3 |
| loss of appetite (16) | 1 | 2 | 3 |
| fluid (water) retention (17) | 1 | 2 | 3 |
| menstrual disorders (18) | 1 | 2 | 3 |

Which of these symptoms bother(ed) you? (Write here the numbers of the symptoms above which bothered you)
